# Multi-Foci Laser Separation of Sapphire Wafers with Partial Thickness Scanning

**DOI:** 10.3390/mi13040506

**Published:** 2022-03-24

**Authors:** Celescia Siew Mun Lye, Zhongke Wang, Yee Cheong Lam

**Affiliations:** 1 SIMTech-NTU Joint Laboratory (Precision Machining), Nanyang Technological University, 50 Nanyang Avenue, Singapore 639798, Singapore; celescia001@e.ntu.edu.sg; 2 School of Mechanical and Aerospace Engineering, Nanyang Technological University, 50 Nanyang Avenue, Singapore 639798, Singapore; 3 Singapore Institute of Manufacturing Technology (SIMTech), A*STAR, 2 Fusionopolis Way, Singapore 138634, Singapore; 4 Genuine Solutions Pte Ltd., 80 Ubi Ave 4, #04-02, Singapore 408831, Singapore

**Keywords:** multi-foci, two-zone partial thickness scanning, sapphire wafer, laser microprocessing, nonlinear absorption

## Abstract

With multi-foci laser cutting technology for sapphire wafer separation, the entire cross-section is generally scanned with single or multiple passes. This investigation proposes a new separation technique through partial thickness scanning. The energy effectivity and efficiency of the picosecond laser were enhanced through a two-zone partial thickness scanning by exploiting the internal reflection at the rough exit surface. Each zone spanned only one-third thickness of the cross-section, and only two out of three zones were scanned consecutively. A laser beam of 0.57 W and 50 kHz pulse repetition rate was split into 9 foci, each with a 2.20 μm calculated focused spot diameter. By only scanning the top two-thirds sample thickness, first its middle section then upper section, a cleavable sample could result. This was achieved with the lowest energy deposition at the fastest scanning speed of 10 mm/s investigated. Although with partial thickness scanning only, counter intuitively, the cleaved sample had a previously unattained uniform roughened sidewall profile over the entire thickness. This is a desirable outcome in LED manufacturing. As such, this proposed scheme could attain a cleavable sample with the desired uniformly roughened sidewall profile with less energy usage and faster scanning speed.

## 1. Introduction

Traditional contact cutting methods, such as a diamond saw blade, result in large kerf widths and debris generation with significant loss of material [1,2,3]. Lasers are seen as a preferred tool to dice brittle materials as they are contactless. Lasers have been engaged in various sapphire wafer cutting techniques such as dual-wavelength double-pulsed laser irradiation [4], laser-induced plasma-assisted ablation [5,6], controlled thermal cracking [7], and filamentation [8,9]. Depending on the laser dicing technique, the surface roughness of sidewalls ranges from 50 nm to 2000 nm [3].

Stealth dicing is a method that focuses the laser into a transparent material, for example glass or sapphire, minimizing the amount of top surface debris generated. For stealth dicing to work in an optically transparent material, an ultrashort pulse laser of sufficiently high intensity is necessary for nonlinear interaction and internal modification of the material. This will minimize the kerf width and debris produced [3,10,11,12].

In conventional stealth dicing methods, the laser light is focused into a single laser spot in the transparent sapphire sample [10,11,12]. These methods employ either single laser scan or multiple laser passes. With a single focus spot, there is an uneven distribution of laser energy within the samples, with rather non-uniform separation sidewall roughness. These sidewall profiles contain both rough laser-modified areas and smooth cleaved surfaces.

Multifocal optics has been employed with promising results to improve the stealth dicing of transparent materials. These include glasses and crystals of different thicknesses. Multi-foci laser irradiation distributes laser energy more evenly over the sample thickness with better controlled dicing. Current multi-foci laser dicing techniques generate multiple foci by employing concave mirrors [13], annular coaxial focusing lenses [14], and diffractive optical elements [15,16]. Allocating laser energy to multi-foci reduces the size of heat-affected zones and the extent of non-uniform distributions of thermal and mechanical stresses in the sample’s thickness, and hence reduces the possibility of crack formation [13,14,15,16]. These methods generally irradiate a transparent medium with a single laser scan only. It could be reasoned that introducing multi-foci for sample dicing could avoid the need for multiple laser passes.

However, if laser power is the limiting factor, integrating multi-foci laser scanning with multiple passes may be necessary. Ma et al. explored the use of Fresnel lenses, a type of diffractive optical element, to separate 2 mm thick sapphire wafers with four passes of multi-foci scanning over the entire thickness [15]. A 1064 nm picosecond laser of 20 W power and 500 kHz repetitive rate was employed to irradiate the samples at 4 mm/s scanning speed. Four scans over four individual 0.5 mm sections were conducted with 15 foci. The laser pulse energy per focus may be calculated to be 2.67 μJ. Spherical aberration had been identified as a significant factor resulting in the reduction of laser energy density of the foci, especially for foci further from the laser source.

When compared with silicon and silicon carbide, sapphire is the most economically viable material as the main substrate in light-emitting diodes (LEDs) [17]. Patterned sapphire substrates (PSS) were introduced to improve the performance of the conventional sapphire substrates [18,19,20,21]. The rough patterned surface of the PSS enhances the LED light extraction by increasing light scattering. The current method of etching the PSS structure into individual sapphire chips before growing the LED layers can be rather time consuming. Industrial-scale production can be expedited by first etching the patterns onto the wafers before singulation. Laser singulation of PSS can be challenging because of the apparent roughened surface. Light emission of LEDs grown on sapphire substrates can be further improved by introducing a roughened sidewall (i.e., the cleavage plane) [11,12,21,22]. It reduces internal reflection within the material. This enables a greater number of photons to escape from the material, which results in an increase in light extraction efficiency in LEDs. However, with non-uniformity in the sidewall profile by a single focus laser scan, the full potential of light emission in LEDs might not be realized [10,11,12].

Our previous studies determined that for laser processing, a sapphire sample with a polished single surface was a better choice than one with both surfaces polished [16,23]. The orientation of the roughened sample, either a laser entry or exit surface, has a significant effect on laser processing of the material. We have determined previously that some of the energy of the high-intensity laser beam is reflected back into the material by internal reflection at the rough exit surface. This partial internal reflection would be diffuse as the micro-surfaces of the rough surface are irregular. This reflected energy is trapped within the sample’s interior and decreases the transmission of light out of the sample. As a result, a significant amount of energy is accumulated within the sample to enhance nonlinear absorption. Nonlinear modification by a sample can facilitate the weakening of the sample sufficiently for its separation. Hence, this partial internal reflection phenomenon at the rough exit surface may be exploited for machining single-surface-polished sapphire samples.

Through multi-foci laser cutting technology for sapphire singulation, we have previously demonstrated that a roughened sidewall profile can be achieved through nonlinear modification at the various focus locations [16]. Sample separation was achieved with the multi-foci laser focused over a single one-third section of the sample thickness. As such, only a portion of the thickness was nonlinearly modified to form a roughened sidewall. This large energy concentration at a single limited section instead of the overall thickness resulted in undesirable large wave-like cracks at the cleavage plane. With the multi-foci laser focused over the whole cross-section of the sample thickness, the roughened sidewall after sample cleavage remained rather non-uniform. Thus, the process requires improvement to achieve the desirable outcome of a uniformly roughened sidewall over the whole thickness of the sample after cleavage.

It is hypothesized here that by exploiting the partial internal reflection of a rough exit surface, the section next to the exit surface need not be scanned to achieve sample cleavage. In addition, as the exit section is furthest away from the laser source, focus next to the exit surface has the most severe spherical aberration [15]. Thus, this approach may have the added benefit of alleviating the deteriorating effect of spherical aberration as laser beam reflection will reinforce its intensity. This hypothesis is verified in this investigation through two-zone partial thickness scanning. Two-zone partial thickness scanning is defined here as the multi-foci laser irradiation with two consecutive scans without scanning the full sample cross-section; each scan is directed at a different section of the sample’s thickness in the same scanning line. It is demonstrated that the desired outcomes can be achieved with effectivity and efficiency improvement of the singulation process by reducing the pulse energy per focus and amount of laser energy deposition required. By studying vastly different scanning speeds over a few orders of magnitudes, the pulse energies required for the desired sample cleavage and sidewall profiles can be estimated.

## 2. Materials and Methods

Single crystal $Al_{2}O_{3}$ sapphire wafers of 50.8 mm in diameter and 0.43 mm in thickness were obtained from Latech Scientific Supply Pte. Ltd. (Singapore). The refractive index of the wafers was 1.768. The wafers were cut into 10 mm wide sample strips for easier handling. The wafers had their single polished surface oriented as the top laser entry surface [23].

A stylus profilometer Form Talysurf Series 2 by Taylor Hobson was employed for surface roughness measurements. To measure the roughness of the sample surfaces (i.e., rough and smooth), the stylus travelled 10 mm across the center of the wafers. The measurements were conducted in two directions (i.e., 0° and 90°); each direction recorded 6 values for a total of 12 values. The averaged measurements in each direction for each sample surface were similar. The surface roughness for each sample surface was concluded to be uniform, without any orientation effect.

The polished surface roughness had Ra = 0.00193 μm (standard deviation 0.00015 μm) and Rz = 0.00980 μm (standard deviation 0.00061 μm). The rough surface roughness had Ra = 0.954 μm (standard deviation 0.059 μm) and Rz = 6.74 μm (standard deviation 0.544 μm). The rough surface roughness was similar to the PSS etching depth (i.e., from 0.4 μm to 1.5 μm) [18,19,20]. Therefore, the roughened surface of the sapphire samples may be used as a proxy for a PSS surface.

For scanning a sample in its c-plane crystal orientation, a 10.3 ps Nd:YAG laser by Time-Bandwidth Products AG (Zurich, Switzerland) was used. With a beam expander, the 1064 nm wavelength laser with vertical linear polarization had a 6.5 mm beam diameter, with an M^2^ value of 1.3. For high peak power and laser intensity, 50 kHz pulse repetition rate was used for a maximum laser power output of 2.4 W. The c-plane crystal-oriented sapphire sample was placed on an automated stage that controlled the laser scanning speed. The sample was scanned perpendicular to the laser’s optical axis.

**Figure 1:**
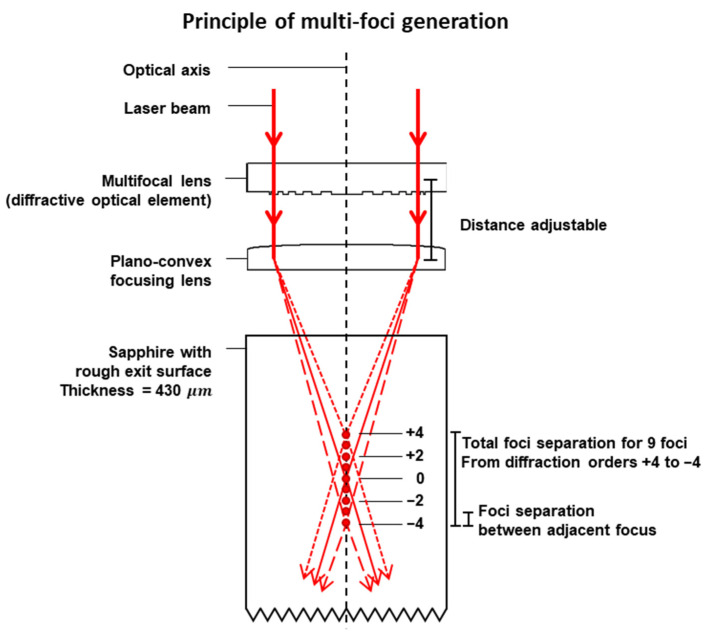
Schematic of 9-foci generation within the sapphire sample.

Two-zone experiments were conducted with a 9 foci multifocal lens (MF lens) and a plano-convex focusing lens with a 7.5 mm effective focal length (EFL). The MF lens is a form of diffractive optical element and the multifocal optics working principle has been previously discussed in [16]. In summary, the MF lens diffracts and changes the phase angle of the incoming laser beam. The laser beam is then focused by the focusing lens to the various foci within the sapphire sample; see Figure 1. The z position was calibrated first with only the plano-convex focusing lens (i.e., without the MF lens) to locate the focal plane of the laser beam. The focal plane of the focusing lens corresponded to the diffraction order 0 in the multi-foci setup. Subsequently, the MF lens was inserted to generate the multi-foci.

To facilitate discussion, the focused spot diameter with or without the MF lens may be assumed to be similar. As such, the focused spot diameter can then be calculated as 2.20 μm at the focal length. Our private communication with the manufacturer indicated that “*the light beams will mostly be the same, but not exact*” [24]. This indicates that although the focused spot diameter may not be exactly the same, it will be similar for all foci. By assuming similar focused spot diameter for all foci, the power density of each focus can be calculated approximately to be 1.65 MW/cm^2^.

This experimental setup had a total foci separation of 136.3 μm, i.e., approximately one-third of the sample thickness (430 μm). Total foci separation is defined as the distance between the first and last foci generated, i.e., the distance between foci of diffraction orders +4 and −4 of this setup. The separation of the foci will differ in sapphire from that in air. This difference has been accounted for through calculations using the equations provided by the manufacturer [24]:

(1) $$\frac{1}{f_{m}}=\frac{1}{f_{R}}+\frac{m}{f_{D}},\;\;m\;=\;\pm 1,\pm 2,\pm 3,\pm 4$$

(2) $$f_{m,n}=nf_{m}$$

where m is the focal length of diffraction order m, $f_{R}$ is the focal length of refractive (focusing) lens, $f_{D}$ is the focal length of diffractive (multifocal) lens, and $f_{m,n}$ is the focal length of diffraction order m in a medium of refractive index n. Focal length values are absolute values measured from the principal plane of the focusing lens. When n=0, $f_{m}=f_{R}$.

For two-zone scanning, two out of the three non-overlapping scanning zones, namely the upper, middle, and lower one-third sections of the thickness, may be selected. Our separate investigation indicated that it is preferable to scan the section further from the laser source first; this is then followed by a second scan on a section closer to the laser source. This ensures that nonlinear modifications, if any, from the first scan would not interfere undesirably with the second scan.

Each scanning zone was labeled with “L”, “M”, or “U” to indicate the lower, middle, or upper one-third section, respectively. Since two consecutive scans were conducted for each sample, the order of the labels indicated the order of the scans conducted. The different two-zone combinations were identified as LM, LU, and MU, respectively. For example, LM means that the lower section is scanned first, and then the middle section is scanned over the same scanning line.

**Figure 2:**
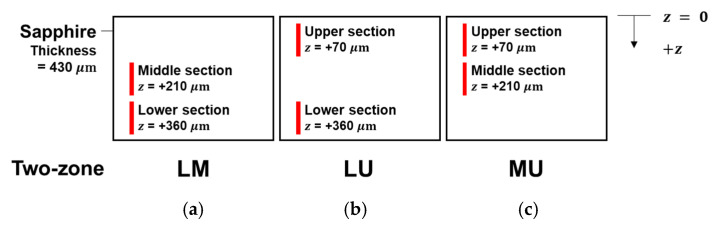
Schematic of two-zone scanning of one-third sections of wafer thickness with middle focus located at the indicated z values for two-zone (**a**) LM (lower-middle sections), (**b**) LU (lower-upper sections), and (**c**) MU (middle-upper sections).

Figure 2 shows the schematic of different two-zone combinations (i.e., LM, LU, and MU). Each vertical solid line signifies all the foci positions for each scanned section. The diffraction order 0 is the middle focus at the mid-point (i.e., at the z values shown) in the vertical column of multi-foci as indicated by the vertical line.

For all experiments, each one-third section was scanned by a 0.57 W laser beam which is approximately a quarter of the maximum laser power output. This is to demonstrate that a key practical limitation due to the laser power constraint could be effectively addressed by this proposed approach. The laser pulse energy per focus was calculated as 1.26 μJ, which was less than half of 2.67 μJ in [15]. The effects of energy deposition within a sample were studied with three scanning speeds, namely 0.1, 1.0, or 10 mm/s. These scanning speeds were chosen to increase by an order of magnitude to cover a large range of scanning speeds and hence pulse energies deposition into a sample. A slower scanning speed would result in more energy deposited; see Table 1. This allows the estimation of the energies required to achieve sample separation with the desired sidewall profiles.

Through stealth dicing, the sample surface was rather clean. The samples were only cleaned with a gentle wipe by a lens cloth before examination under Olympus BX51 fluorescent microscope. After laser irradiation, the samples were mechanically cleaved. For samples that could be separated, they can be easily cleaved by hand with a normal force applied along the laser-scribed lines to aid the propagation of cracks for sample separation. If the samples failed to separate or the plane of separation deviated from the intended separation plane (i.e., laser-scribing plane), “no cleavage” was recorded for the sample. Indeed, for those samples that failed to separate, an increase in the normal force might result in large separation deviation from the intended separation plane, or the sample remained intact. The sidewall profiles of successfully cleaved samples were examined with a confocal laser scanning microscope VK-110 by Keyence.

## 3. Results

### 3.1. Top Polished Entry Surface for Two-Zone Scanning

**Figure 3:**
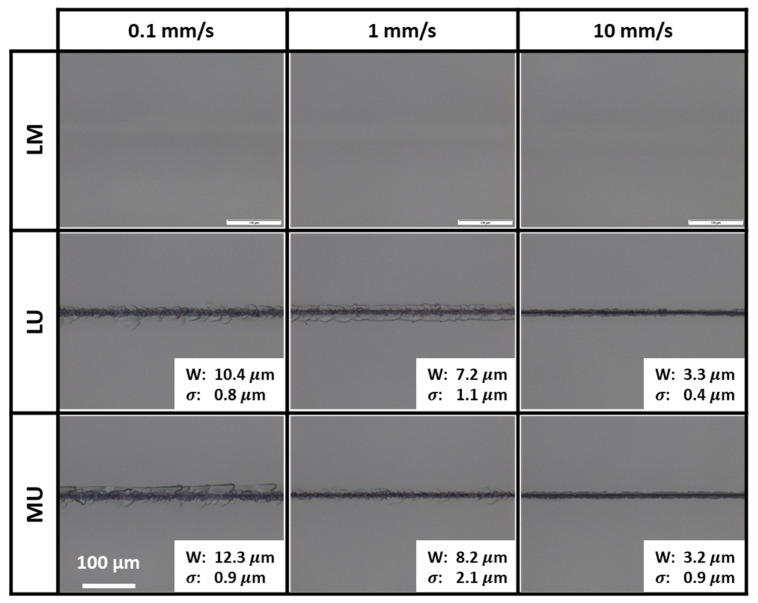
Top polished entry surface optical images for two-zone scanning showing top surface scribes (if any) with their widths (W) and standard deviations (σ) indicated.

The top polished entry surface optical images of the samples after two-zone laser scanning with different speeds are shown in Figure 3. It presents the top surface scribes (if any) with their widths and standard deviations indicated. The widths were extracted using a calibrated optical microscope. The standard deviations were calculated from three measurements. Only the width of the scribes without the crack was considered.

#### 3.1.1. Scribe Formation with Two-Zone LM (Lower-Middle Sections)

As shown in Figure 3, since the second scan of LM was conducted in the middle section, for all scanning speeds only subsurface damages were detected on the top polished entry surface as expected. The significant distance between the middle section and the top surface did not allow top surface scribe formation. Any internal modifications or cracks formed were mainly confined within the middle and lower sections.

#### 3.1.2. Scribe Formation with Two-Zone LU (Lower-Upper Sections)

The proximity of the second scan of LU at the upper section resulted in top polished entry surface scribes. As indicated in Figure 3, an increase in scanning speed caused a decrease of energy deposition and a reduction of scribe widths on the top polished surface.

At the lower scanning speeds of 0.1 and 1.0 mm/s, top polished surface crack-dominated scribes surrounded by small cracks were detected. These scribes were formed primarily by the large laser energy deposition. With 0.1 mm/s scanning, the top surface cracks detected had shorter cracks that did not greatly deviate from the scanning direction.

With the fastest scanning at 10 mm/s, a top polished surface ablation-dominated scribe was detected. By intuition, with less energy deposition, one might expect that no scribes should be formed. However, the more desired ablation scribes were formed instead. With less energy deposited, the lack of large amounts of excess energy could not form or propagate internal cracks to the top surface. Instead, the energy was utilized mostly for upper section internal modifications and top surface scribe ablation.

#### 3.1.3. Scribe Formation with Two-Zone MU (Middle-Upper Sections)

For samples scanned with MU, there was formation of the top surface scribes as the upper section scan was close to the sample’s top surface; see Figure 3. With slower scanning at 0.1 and 1.0 mm/s, a top surface crack-dominated scribe was formed. With the fastest scanning at 10 mm/s, a top surface ablation-dominated scribe was formed. The top surface scribes were formed similarly to those in LU.

With 0.1 mm/s scanning, the top surface cracks detected seemed to be longer, with deviation from the scanning direction as compared to that of LU; see Figure 3. For LU, the distance between the lower and upper scanning sections was large. This minimized the interaction between the second scan laser beam in the upper section and the modified area formed by the first scan in the lower section. As such, the energy from the second scan of LU only caused a thicker crack-dominated scribe; there was inadequate energy for crack elongation on the top surface. In contrast, for MU, although the samples also had scans in the upper section as in LU, the middle and upper scanning sections were next to each other. This facilitated the interaction between the second scan laser beam in the upper section and the nonlinearly modified area of the first scan in the middle section. The modified area likely acted as micro-internal surfaces. They could have reflected the second scan laser back towards the top surface and intensified the amount of energy of the upper section. With more high-intensity energy accumulated at the upper section, a thick crack-dominated scribe was formed, with crack elongation that deviated further from the scanning line. As such, these long and deviated top surface cracks could be observed for MU scanning but not for LU scanning.

### 3.2. Bottom Rough Exit Surface Scribes

**Figure 4:**
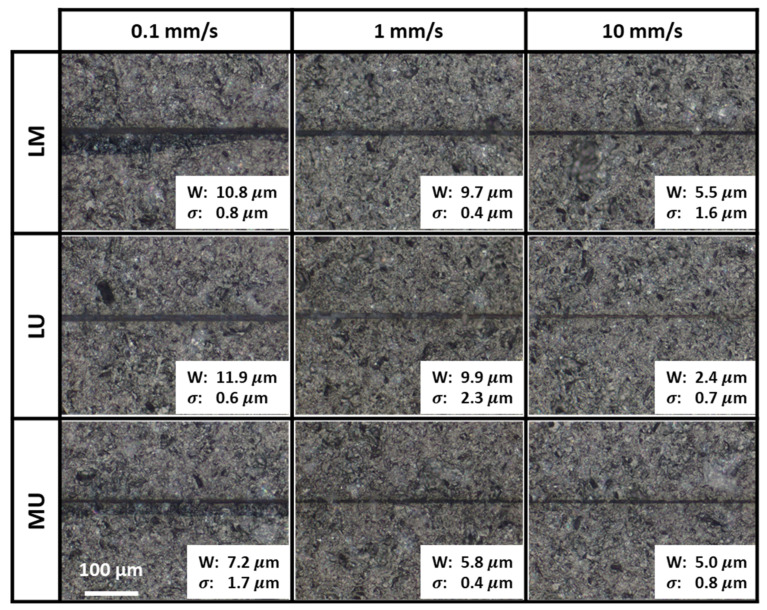
Bottom rough exit surfaces optical images for two-zone scanning showing bottom surface scribes with their widths (W) and standard deviations (σ) indicated.

Bottom rough exit surface optical images for different two-zone scanning with different scanning speeds are shown in Figure 4. It presents the bottom surface scribes with their widths and standard deviations indicated. The widths were extracted using a calibrated optical microscope. The standard deviations were calculated from three measurements. Only the width of the scribes without the crack was considered.

#### 3.2.1. Scribe Formation with Two-Zone LM (Lower-Middle Sections)

The lower section was scanned first by LM. As the lower section was close to the bottom surface, bottom surface ablation-dominated scribes were detected as expected for all scanning speeds; see Figure 4. Partial internal reflection, likely with surface light scattering, could have been enhanced by the bottom rough exit surface. Thus, instead of being transmitted out of the sample, the laser beam was reflected back into the sample; its energy was trapped effectively at the vicinity of the rough exit surface. With the incoming beam reinforced by the partially internally reflected beam, an ablation scribe was formed when there was sufficient energy accumulation [16,23]. An increase in the scanning speed would have resulted in a reduction of energy deposition, and thus the reduction of the width of the ablation scribes as indicated in Figure 4.

With 0.1 mm/s scanning, an offset crack adjacent to the ablation scribe was observed for LM. After the first lower section scan, the nonlinear modifications functioned as micro-surfaces, hindering the laser beam propagation along the optical axis. The proximity of the second middle scanned section facilitated the interaction between the second scan laser beam and the first scan nonlinear modifications. As such, part of the laser beam was likely reflected upwards, interacting with the incoming beam. This further intensified and accumulated energy within the sample. Internal cracks were formed, propagating to the bottom surface by the excess energy. The crack was offset to the ablation scribe on the bottom surface as the crack propagation was not controlled. Therefore, an offset crack was detected next to the ablation scribe for the sample scanned with LM.

#### 3.2.2. Scribe Formation with Two-Zone LU (Lower-Upper Sections)

As LU first scanned the lower section close to the sample’s bottom surface, bottom surface scribes were detected for all scanning speeds; see Figure 4. The bottom surface ablation-dominated scribes were formed in a similar manner as those in LM. An increase in scanning speed caused a reduction of energy deposition and a reduction of the scribe width on the bottom surface as indicated in Figure 4.

With 0.1 mm/s scanning, only an ablation scribe without an offset crack was observed for LU; see Figure 4. This is in contrast with the observation of an offset crack adjacent to the ablation scribe for LM. For LU, the large distance between the lower and upper sections minimized the interaction between the two scans. As such, the formation of the bottom surface scribe was caused only by the lower section scan.

#### 3.2.3. Scribe Formation with Two-Zone MU (Middle-Upper Sections)

No bottom surface scribes should be observed with MU scans due to the large distance between the middle section and the bottom surface. Yet, counter intuitively, bottom surface scribes could be detected; see Figure 4. The width of the bottom surface ablation-dominated scribes decreased with increasing scanning speed as indicated in Figure 4.

On closer examination, partial internal reflection of the laser beam by the bottom rough exit surface could well result in ablation scribe formation by the first scan in the sample’s middle section. This occurred despite the substantial distance between the middle section and the bottom surface. The reflected laser beam led to an increase in laser intensity at the sample’s lower section, similar to that observed in [13] with the aid of concave mirrors. The reinforcement of the incoming beam by the partially internally reflected beam resulted in adequate trapping of high-intensity laser energy in the region, thus forming the ablation scribes observed [23].

In particular, at the slowest 0.1 mm/s scanning, the excess energy deposited caused the propagation of the internal cracks to the bottom surface, thus forming the offset crack observed, similar to that by LM. With the associated energy deposition reduction for the faster scanning at 1.0 and 10 mm/s, the available energy was insufficient for the formation and propagation of internal cracks (if any) to the bottom surface.

### 3.3. Sample Separation and Sidewall Profiles

For different two-zone laser scanning with different scanning speeds, Table 2 summarizes if the sample could be successfully cleaved.

#### 3.3.1. Unsuccessful Sample Separation with Two-Zone LM and LU

LM-scanned samples could not be cleaved regardless of scanning speeds. The ineffective nonlinear modifications within the samples could not induce a complete separation crack throughout the sample thickness during the cleaving process. These experiments demonstrated that LM was not an ideal configuration to achieve sample separation even though bottom surface scribes were formed; see Figure 4.

From the top and bottom surface optical images of LU-scanned samples, it was observed that scribes were formed on both surfaces; see Figure 3 and Figure 4. By intuition, it is expected that a sample scanned with LU would have produced damaged lower and upper sections that were more prone to cleavage. The initial crack at these sections should have propagated across the thickness and should have assisted in the complete separation of the sample. However, with a rather large separation of these two scans, the unscanned middle section had retained its integrity. The samples were insufficiently weakened with unsuccessful cleavage for LU scanning. Therefore, these experiments demonstrated that the presence of scribes on both surfaces of the sample was not a necessary indication of successful sample cleavage.

#### 3.3.2. Sample Separation with Two-Zone MU (Middle-Upper Sections)

**Figure 5:**
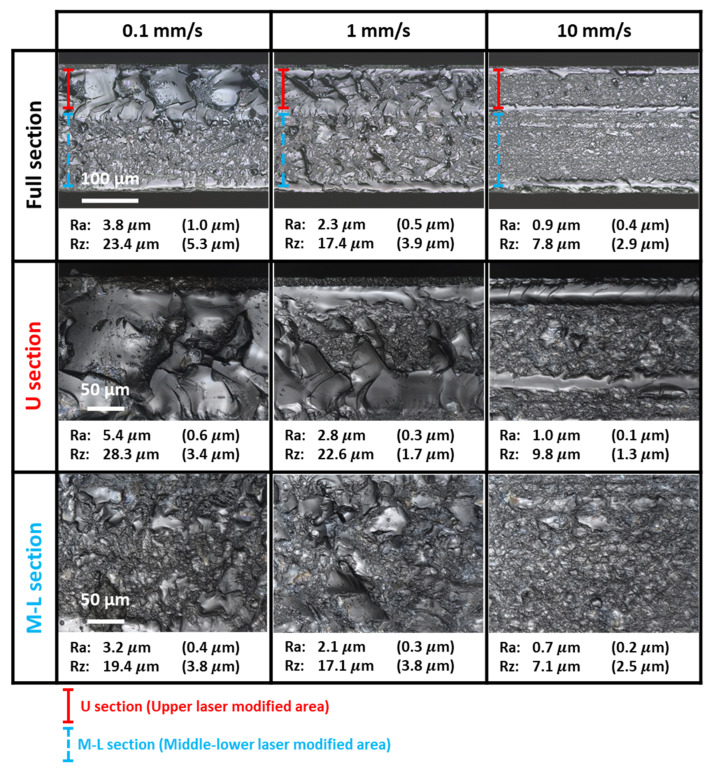
Sidewall profile laser-scanned images of cleaved MU samples at higher magnifications and roughness measurements Ra and Rz (with standard deviations). The solid red line indicates the upper laser-modified area; the dashed blue line indicates the middle-lower laser-modified area.

In contrast, with scribes observed on both surfaces, samples scanned with MU were successfully cleaved regardless of the scanning speed; see Table 2. In addition, since only the middle and upper sections were scanned, nonlinear modification of the sample should only occur in the top two-thirds of the thickness. Instead, counter intuitively, the sidewalls were observed to be roughened more frequently and uniformly over the entire thickness of the sample; see Figure 5 for magnified laser-scanned images with surface roughness measurements. The surface roughness of the sidewall was measured with a confocal laser scanning microscope VK-110 by Keyence. Ten horizontal roughness measurements for each section were recorded and their averages and standard deviations are presented. Full section roughness was measured spanning over both the upper and middle-lower sections.

**Figure 6:**
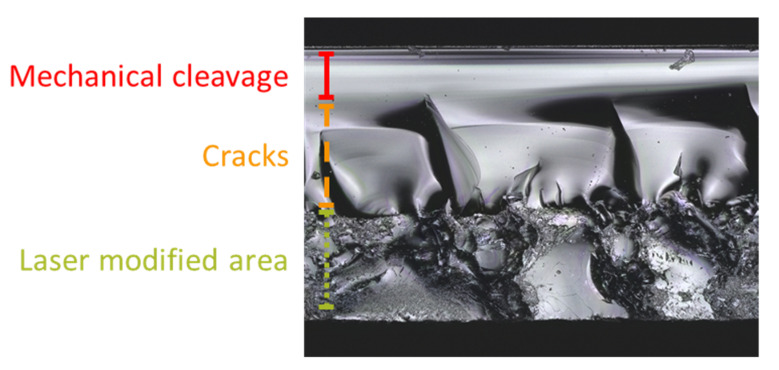
Sidewall profile showing mechanical cleavage, cracks, and laser-damaged areas (adapted from [16]).

For proper identification of the various areas, these observations in Figure 5 were cross-referenced with a representative sidewall image of our previous investigation on multi-foci laser dicing of sapphire wafers [16]. Figure 6 shows the sidewall profile by a 50 kHz 0.8 W laser with 27 foci and a 4.38 mm EFL objective lens scanning only the middle section at 1 mm/s scanning speed. Visually, separation due to mechanical cleavage resulted in a smooth sidewall; its cracked areas had much higher surface roughness with wave-like structures. In contrast, the laser-damaged areas resulted in a roughened sidewall. The roughness measurements of the cleaved surface of the sample in [16] are presented in Table 3. Compared to our previous investigation in Figure 6, both visually and in roughness measurements, the sidewall images of the cleaved samples of this investigation in Figure 5 consist mostly of laser-damaged areas with some cracked and mechanical cleavage areas presented in some sidewalls. These sidewall profiles in Figure 5 warrant more detailed examination.

For MU scans, two distinct laser-modified areas were identified, namely an upper laser-modified area and a middle-lower laser-modified area (i.e., in the middle and lower sections). There were gaps where there was no visible laser modification on the sidewall. These gaps contained either wave-like cracks from excess energy deposited or were smooth due to mechanical cleavage. Smaller laser-modified areas at both top and bottom surfaces substantiated the type of scribe formation on the surfaces.

Caused by the high-intensity laser beam of the second upper section scan, the upper laser-modified area was formed through nonlinear absorption at the various multi-foci spots, resulting in the formation of a roughened sidewall. Although the roughened sidewall observed was mainly formed by nonlinear modifications, there could be small internal cracks formed by the surplus in energy deposition.

The middle-lower modified zone appeared to consist of both the middle and lower sections, despite only the middle section being scanned. Indeed, hardly any distinction could be observed between these two sections. This is verified by the similar standard deviation of the roughness value for the combined middle and lower sections as compared to that for the upper section. This observation can be ascribed to the bottom rough exit surface partial internal reflection (previously discussed in the formation of the bottom surface scribes in Section 3.2). Therefore, the two scans produced a cross-section of laser modification along the optical axis across the entire sample thickness. The laser modification formed by scans of MU effectively weakened the sample for cleavage. It should be highlighted that without the rough exit surface to enhance the occurrence of partial internal reflection, the lower section would not be nonlinearly modified.

Increasing scanning speed resulted in a decrease in energy deposited. Less excess energy was available for wave-like internal cracks formation in the sidewall profile. The amount of energy deposited determined the size and frequency of the wave-like patterns and the uniformity and frequency of the distortions formed by nonlinear modification. Large excess energy deposited resulted in large and more wave-like patterns of internal cracks with low uniformity and frequency of the nonlinear modified distortions. At the slowest scanning speed investigated (0.1 mm/s), the combination of internal cracks and laser-modified areas resulted in the roughest sidewall observed with large standard deviations; see Figure 5. With a smaller amount of deposited energy at the fastest scanning speed investigated (10 mm/s), it was just sufficient to form internal modification within the sample. Smaller and less wave-like patterns of internal cracks were observed; this resulted in an increase of uniformity and frequency of the internal modifications.

In our previous investigation [16], sample separation could not be achieved by multi-foci irradiation of a single section with the same total laser power and amount of energy deposition. However, with MU, not only could the samples be separated, but a desired uniform roughened sidewall profile was also achieved with less energy usage, i.e., at the fastest scanning of 10 mm/s investigated. This implies that more efficient production can be achieved with a higher rate of scanning. This demonstrates the superiority of employing two-zone MU scans for sample separation.

Table 4 summarizes the sidewall roughness of cleaved sapphire samples irradiated by different laser dicing techniques and parameters. These techniques include Bessel beam irradiation, single focus laser dicing, multi-foci laser dicing with full or partial thickness irradiation, and single or multiple section scanning. The sidewall roughness values of the cleaved sapphire samples could differ by more than one order of magnitude, and some had rather non-uniform sidewall profiles. Current investigation could achieve rather uniform sidewall roughness; the roughness values were at the mid-range of the roughness values presented in Table 4. This indicates the potential of fine tuning the operational parameters to increase or decrease the sidewall roughness as required.

### 3.4. Spherical Aberration and Internal Reflection at the Exit Surface

**Figure 7:**
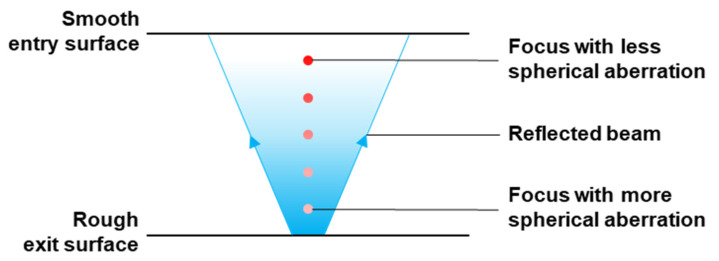
Interaction between the reflected beam and multi-foci with spherical aberration.

These results indicate that the deteriorating effect caused by spherical aberration could well be alleviated by exploiting the partial internal reflection of the laser beam at the bottom rough exit surface [23]; see Figure 7. The focusing imperfection with a decrease in laser energy intensity caused by spherical aberration becomes more severe at locations closer to the exit surface and further away from the laser source [15]. In contrast, the reflection of the laser beam is hypothesized to increase the laser power intensity most effectively at the vicinity of the rough exit surface. Its effect will be less further away from the exit surface. This trend is exactly opposite to that caused by spherical aberration. Indeed, this investigation indicates that the effect of beam reflection at the exit surface is significant. The one-third section next to the exit surface was not scanned and yet the samples showed clear laser damage at the cleaved surface of this one-third section. This was likely due to the increase in laser intensity at this section through partial internal reflection of the laser beam.

In contrast, for LU scanning or LM scanning, where the middle or the top one-third section was not scanned, cleavage of the samples was not successful. It is speculated that this is because the reflected beam had much less effect at the middle or the top one-third section, which was rather far away from the exit surface.

## 4. Conclusions

Multi-foci laser cutting technology has been adapted for machining transparent sapphire wafers [15,16]. This investigation proposed an approach of employing two-zone partial thickness scanning for machining a single-surface polished sapphire sample. Cleavable samples were achieved with two-zone MU scanning. It scanned only the top two-third thickness by first scanning the middle section, then the upper section. The upper section scan resulted in nonlinear modifications in the upper section. The middle section scan formed nonlinear modifications in both the scanned middle and non-scanned lower sections. Despite this partial thickness scanning, the cleaved samples had a uniformly roughened sidewall profile over the entire thickness. This is a desirable outcome in LED manufacturing.

It should be noted that scanning either the upper one-third and lower one-third sections (i.e., two-zone LU) or the lower two-third sections of the sample thickness (i.e., two-zone LM) did not result in sample cleavage. By comparing MU, LU, and LM scanning, it is further hypothesized that the effect of beam reflection at the rough exit surface is significant. The beam reflection could well alleviate the focusing imperfection caused by spherical aberration. More importantly, there could be an increase in laser intensity at the non-scanned section in MU through partial internal reflection of the laser beam. It provides a plausible explanation that in MU scanning, the non-scanned one-third section next to the exit surface showed clear laser damage at the cleaved surface. However, we will not rule out that there could well be other possible explanations for our observations.

This technique of partial thickness two-zone MU scanning resulted in a cleavable sample with the desirable uniformly roughened sidewall profile. Quality outcomes were achieved with less energy usage and a faster scanning speed, and thus an improvement of laser energy effectivity and efficiency. Although double scans have been demonstrated in this investigation, it does indicate the possibility of increasing the number of scans and scanning sections for a thicker sample if necessary to achieve sample separation. The laser power utilized is approximately a quarter of the total power of the laser system. This provides much room to increase the laser power with more foci over a larger total foci separation distance for a thicker sample. This investigation can be further extended to other materials such as the laser singulation of single-surface polished silicon wafer with a rough surface as the exit surface. However, optimization of the various process parameters will be necessary for the specific applications as required.

In addition, for a better understanding of the exact mechanism, a thermal camera may be employed to quantitatively evaluate the interaction of the reflected laser beam on the multi-foci of the incoming beam at the various one-third sections. A comparison between the results of single-surface and double-surface polished samples could explain the effects of beam reflection and its effectiveness in reducing the problems caused by spherical aberration.

## Figures and Tables

**Table 1:** Laser parameters for each scanning speed.

Scanning Speed (mm/s)	Energy Deposited at a Single Spot (mJ)	Pulse Overlap
0.1	12.5	1100
1	1.25	110
10	0.13	11

**Table 2:** Sample cleavage for various scanning zones and speeds.

Scanning Zones	Scanning Speed (mm/s)		
	0.1	1	10
LM	No cleavage	No cleavage	No cleavage
LU	No cleavage	No cleavage	No cleavage
MU	Sample cleaved	Sample cleaved	Sample cleaved

**Table 3:** Roughness measurements for sidewall of cleaved sample shown in Figure 6 [16].

Feature	Ra (μm)		Rz (μm)	
	Average	Std. Dev	Average	Std. Dev
Mechanical cleavage	0.5	0.3	2.4	1.6
Cracks	9.0	0.7	40.5	1.7
Laser-modified area	2.6	0.4	20.6	3.7

**Table 4:** Comparison of sidewall roughness of cleaved sapphire samples by various laser dicing methods.

Laser Dicing Method	No. of Foci/Pass	Scanning Speed (mm/s)	Sample Thickness (μm)	Scanned Section (μm)	Sidewall Roughness (μm)		Uniformity of Sidewall
					Ave	Std. Dev	
Bessel beam [3]	-	0.1–7	380, 1000, 1500	Full thickness	0.2–1.3	-	-
Femtosecond laser ablation [25]	1	2	~339	-	4.13	-	Visually uniform
Partial thickness multi-foci dicing (previous work) [16]	15	1	430	136 (1 section scanned)	2.6	0.4	Mechanical cleavage, cracks, non-uniform laser modification
Full thickness multi-foci dicing [15]	21	4	2000	Full thickness	4.5	-	Mechanical cleavage, non-uniform laser modification
Full thickness multi-foci dicing [15]	21	4	500	Full thickness	3.2	-	Visually uniform
Sectional multi-foci dicing [15]	15	4	2000	500 (4 sections scanned)	2.5	-	No micro-cracks reported
Partial thickness multi-foci dicing (current work)	9	10	430	136 (2 sections scanned)	0.9	0.4	Mechanical cleavage, visually uniform laser modification

## Data Availability

Not applicable.
